# Peter Campbell Clifford

**Published:** 1989-11

**Authors:** P W J Houghton


### Obituary—Peter Campbell Clifford, TD, MD MBChB (Bristol) FRCS

Mr P C Clifford, consultant in general and vascular surgery at Frenchay Hospital, Bristol died suddenly on 21st February 1989 aged 39.

Peter Campbell Clifford was born in December 1949 in Ruislip, Middlesex and was educated at the Royal Masonic School in Bushey. He studied medicine at Bristol University graduating MB ChB in 1973. After house jobs at Frenchay and Southmead hospitals he was appointed pathology demonstrator at the Bristol Royal Infirmary. Following this he was a Senior House Officer and Registrar at Swindon and the Middlesex Hospital, London and became a Fellow of the Royal College of Surgeons of England in 1978. He returned to Bristol as a British Heart Foundation Fellow where he developed his interest in vascular surgery whilst studying the techniques of non-invasive blood flow monitoring in the vascular studies laboratory at the Royal Infirmary. For this work he was awarded a Doctorate of Medicine from the University of Bristol in 1982.

From Bristol he moved to Southampton to take up the post of Senior Registrar to the Wessex region. A sabbatical year in 1983 at the Groote Schuur Hospital, Cape Town, enabled him to gain unique experience in vascular trauma. Returning to Southampton he continued to publish prolifically contributing widely to the surgical literature. With his own reserach grants and interests in surgical video production he was also able to offer support and encouragement to other aspiring surgical trainees. He was enormously proud to have been appointed to a Consultant post back in Bristol and was looking forward to his job with great relish. It was therefore particularly ironic that he died 3 weeks after taking up his post.

Peter was a man of great energy refusing to let a single blade of grass grow underfoot. As well as his surgical interests he was a keen off-shore sailor and shot. He was a major in the Territorial Army, serving with both the 4th Battalion The Royal Green Jackets and the Royal Army Medical Corps and was awarded the Territorial Decoration in 1987. From student days he had been an excellent organizer and he produced successful Christmas revues year after year raising many thousands of pounds for charity. With his father also dying at a young age from heart disease it was as if Peter intended to pack as much into his life as he possibly could. In this he most certainly succeeded and his premature death was not just a great loss for Bristol and vascular surgery but also for his large number of friends.

P W J HOUGHTON

